# The Impact of Androgen Receptor Expression on Breast Cancer Survival: A Retrospective Study and Meta-Analysis

**DOI:** 10.1371/journal.pone.0082650

**Published:** 2013-12-04

**Authors:** Qing Qu, Yan Mao, Xiao-chun Fei, Kun-wei Shen

**Affiliations:** 1 Department of Oncology, Ruijin Hospital, Shanghai Jiao Tong University School of Medicine, Shanghai, China; 2 Comprehensive Breast Health Center, Ruijin Hospital, Shanghai Jiao Tong University School of Medicine, Shanghai, China; 3 Department of Pathology, Ruijin Hospital, Shanghai Jiao Tong University School of Medicine, Shanghai, China; Health Canada and University of Ottawa, Canada

## Abstract

Recent studies have highlighted the role of androgen receptor (AR) as a prognostic biomarker of breast cancer. However, its predictive role in disease free survival (DFS) and overall survival (OS) still remains inconclusive. The present study aimed to retrospectively investigate the association between AR and survival outcomes in breast cancer and also identify this association by a meta-analysis of published researches. Clinical data from 109 patients with breast cancer, who underwent surgery at Ruijin Hospital, Shanghai, were retrospectively analyzed for immunohistochemical AR expression measured by tissue microarray. For meta-analysis, articles available in Pubmed on the relationship between AR and breast cancer outcomes were included. Data obtained from both were combined and analyzed. Women with AR positive tumors in the retrospective study had a significantly better DFS (HR 0.24, 95% CI 0.07-0.88) and OS (HR 0.19, 95% CI 0.04-0.85) than women with AR negative ones. Meta-analysis showed that AR expression in breast tumors was an indicator of better DFS (HR 0.52, 95% CI 0.43-0.64). In subgroup analysis, AR could predict DFS outcome in estrogen receptor (ER) positive (HR 0.45, 95% CI 0.34-0.59), ER negative (HR 0.42, 95% CI 0.26-0.67), and triple negative breast cancer (HR 0.40, 95% CI 0.23-0.69). Moreover, in ER positive breast cancer patients, the expression of AR could predict better OS (HR 0.39, 95% CI 0.19-0.82). The present analysis indicated that AR expression was associated with lower risk of recurrence in patients with all breast cancer types and better OS in cases with ER positive.

## Introduction

Breast cancer is one of the most common malignancies among women all over the world. It can be classified into five subtypes based on molecular therapy: luminal A , luminal B, normal breast-like, basal-like, and human epidermal growth factor receptor 2 (HER2) overexpressing tumors[1]. Different subtypes require different therapeutic strategies. To date, estrogen receptor (ER), progesterone receptor (PR), and HER2 have been proved to be important prognostic indicators for breast cancer. More importantly, they are also essential in determining the use of hormone therapy, chemotherapy, and targeted therapy in different subtypes. For example, the presence of hormone receptors including ER and PR could suggest the sensitivity of a tumor to endocrine therapy. In early breast cancer, adjuvant endocrine therapy of tamoxifen (TAM) for 5 years could reduce the risk of death and recurrence by 30 to 40% in hormone receptor positive patients[2]. Recently, adjuvant treatment with trastuzumab has been shown to significantly improve outcomes in patients with HER2-positive breast cancer[3]. Determination of HER2 status has been a standard nowadays for every patient with breast cancer to select adjuvant targeted treatment with trastuzumab. Despite tremendous efforts to reduce metastasis and deaths due to breast cancer, the prognosis is still poor. More than 20% of patients with early breast cancer could eventually develop incurable metastatic disease[2,4]. Therefore, it is high time to identify new targets and biomarkers to improve the prognosis of breast cancer.

Like ER and PR, androgen receptor (AR) also belongs to steroid nuclear receptor family. AR has been an important target in prostate cancer, and it has recently been considered as a potential biomarker in breast cancer. AR is commonly expressed in ductal cancer in situ and invasive breast carcinoma[5]. Moreover, AR can be co-expressed with ER and PR, approximately 60% of the time[6]. 

Roles of AR in breast cancer development and progression have not been very clearly understood. Some researchers have reported that the expression of AR is associated with a better prognosis. But the prognostic significance varies with the different molecular subtypes of breast cancer. To understand the role of AR in disease free survival (DFS) and overall survival (OS), the present study aimed to retrospectively investigate the association between AR and survival outcomes in breast cancer and also to identify this association by a meta-analysis of published researches.

## Methods

### 1. Methods of retrospective Study

#### 1.1. Ethics statement

This study was conducted after approval from the institutional review board of Ruijin Hospital, Shanghai, China; which also waived the need for consent, since there was no interaction with patients enrolled. The study was conducted based on their available medical information, which used in a de-identified fashion.

#### 1.2. Patients and methods

Clinical data of 109 Chinese patients with breast cancer were collected from Comprehensive Breast Health Center, Ruijin Hospital, Shanghai Jiaotong University School of Medicine. All patients received surgical treatment for breast cancer between 2003 and 2008, and they were followed up from the date of diagnosis until May, 2013 or death, whichever came first. The median follow up time was 6.2 years. The characteristics of the patients are below (Table 1). TNM disease stage was classified according to the American Joint Committee on Cancer, 7th Edition.

**Table 1:** Characteristics of patients.

Characteristics number	n=109 (%)
Age (years, mean ± SD)	58±13
T-stage	
T1	55 (50.4)
T2	49(45.0)
T3	2(1.8)
T4	3(2.8)
N-stage	
0	73(67.0)
1	20(18.3)
2	11(10.1)
3	5(4.6)
Histologic type	
IDC	46(42.2)
ILC	49(45.0)
DCIS	14(12.8)
ER status	
positive	79(72.5)
negative	30(27.5)
PR status	
positive	67(61.5)
negative	42(38.5)
HER2 status	
positive	8(7.3)
negative	101(92.7)

IDC, invasive ductal carcinoma; ILC, invasive lobular carcinoma; DCIS, ductal cancer in situ; ER, estrogen receptor; PR, progesterone receptor; HER2, human epidermal growth factor receptor 2.

Archived formalin-fixed paraffin-embedded breast cancer blocks were collected for all patients, and a tissue microarray (TMA) was constructed. Immunohistochemical (IHC) staining of AR, ER, PR, and HER2 was performed. Anti-ER antibody, anti-PR antibody, and anti-AR antibody were obtained from DAKO (Carpinteria, USA) at 1: 100 dilutions. Anti-HER2 antibody was from ROCHE (4B5, Ventana). Sections were considered AR, ER, and PR positive, when >1% of tumor cell nuclei stained positive (Figure S1). HER2 was scored as 0, 1+, 2+, and 3+ according to the American Society of Clinical Oncology/College of American Pathologists guidelines. HER2 negativity was considered as HER2 0, 1+, and 2+; HER2 positivity was considered as 3+.

#### 1.3. Statistical analysis

SPSS 16.0 statistical software (SPSS Inc., Chicago, USA) to perform the statistical analysis to identify prognostic significance of AR. Kaplan–Meier plot and Cox regression analysis were used, and P value of <0.05 was considered significant. DFS was considered as the interval (in months) between the date of breast surgery to first recurrence (locoregional recurrence and/or distant metastasis). OS was defined the time (in months) between the date of breast surgery and time of breast cancer-related death.

### 2. Methods of meta-analysis

#### 2.1. Data selection

PubMed was searched to identify primary research publications on the association between AR and breast cancer prognosis. The following searches terms were used: “breast cancer” and “androgen receptor”. Inclusion criteria of studies searched were: (1) studies which measure AR expression by IHC in breast cancer tissue (2), studies which provide information on DFS or OS comparing AR positive with AR negative patients groups, and (3) articles published in English. The following were considered as exclusion criteria of the search: (1) review or case report, (2) AR was not evaluated by IHC, and (3) lack of key information for hazard ratio (HR) of DFS or OS. When an individual author published several articles obtained from the same patient population, only the newest or most complete article was included in the analysis. After extensive research, a total of 11 original articles were considered for the meta-analysis. 

#### 2.2. Data extraction and methodological assessment

Articles included were assessed independently by two reviewers (Qing Qu and Yan Mao). Data retrieved from the reports included author, journal, year of publication, cut-off value, number of patients, hormone receptor status, HR, and 95% confidence interval (CI) of survival.

#### 2.3. Statistical methods

Statistical variables such as HR and corresponding 95% CI were directly taken and used to combine the data, if they were described in articles. LogHR values were used for aggregation of survival results. A meta-analysis on AR expression was performed both for OS and DFS. Forest plots were used to estimate the effect of AR expression on survival outcomes. I^2^ value was used to evaluate heterogeneity (I^2^ = 0-50%, no or moderate heterogeneity; I^2^ > 50%, significant heterogeneity); and fixed-effect model was used, if there was no significant heterogeneity. Otherwise, random-effect model was used. By convention, an observed HR <1 implied better survival for the group with AR expression. All statistical analyses were conducted using STATA version 11.0 (Stata Corporation, College Station, TX, USA).

## Results

### 1. Results of TMA

Of the 109 patients with breast cancer, 52 (47.7%) were AR-positive and 57(52.3%) were AR-negative (Table S1). Overall, there were 13 deaths (2 deaths in AR-positive and 11 in AR-negative groups) and 15 recurrences (3 recurrences in AR-positive and 12 in AR-negative groups) at the end of follow-up period. 

Cox regression analysis showed that women with AR positive tumors had a significantly better DFS and OS compared with women with AR negative tumors. In univariate analysis, expression of AR was found to be associated with better DFS (p= 0.026) and OS (p= 0.022) (Table 2). In multivariate analysis, adjusted HRs of AR for DFS were 0.244 (95% CI, 0.068–0.876, p= 0.031), and 0.188 (95% CI, 0.041–0.855, p= 0.031) for OS (Table 3).

**Table 2:** Univariate analyses of DFS and OS in all population.

Variables	DFS		OS	
	HR (95% CI)	p	HR (95% CI)	p
Age, years (>40 vs ≤40)	0.903 (0.119-6.872)	0.922	0.788 (0.102-6.068)	0.819
Stage (III vs I/II)	1.549 (1.132-2.118)	**0.006**	1.568 (1.131-2.175)	**0.007**
Receptor status (pos. vs neg.)	0.446 (0.159-1.255)	0.126	0.495 (0.162-1.515)	0.218
AR status (pos. vs neg.)	0.237 (0.066-0.843)	**0.026**	0.171 (0.038-0.776)	**0.022**

DFS, disease free survival; OS, overall survival; CI, confidence interval ; HR, hazard ratio; AR, androgen receptor.

**Table 3:** Multivariate analyses of DFS and OS in all population.

Variables	DFS		OS	
	HR (95% CI)	p	HR (95% CI)	p
Age, years (>40 vs ≤40)	1.092 (0.127-9.405)	0.936	1.128 (0.123-10.331)	0.915
Stage (III vs I/II)	1.604 (1.122-2.293)	**0.010**	1.594 (1.092-2.327)	**0.016**
Receptor status (pos. vs neg.)	0.394 (0.135-1.148)	0.088	0.431 (0.135-1.374)	0.155
AR status (pos. vs neg.)	0.244 (0.068-0.876)	**0.031**	0.188 (0.041-0.855)	**0.031**

DFS, disease free survival; OS, overall survival; CI, confidence interval ; HR, hazard ratio; AR, androgen receptor.

### 2. Results of meta-analysis

A total of 1376 potentially relevant citations were reviewed. Through extensive research, a total of 12 articles, including 11 published articles and the present retrospective study, were included in the meta-analysis (Table 4)[7-17]. All the included articles were on the association of AR expression with DFS and/or OS and covered a total of 5270 patients with breast cancer. Five articles reported the HR of AR expression in patients with ER positive breast cancer in text or table, while six articles reported the HR in patients with ER negative breast cancer.

**Table 4:** Main characteristics and results of the eligible studies.

Author	Year	Country	Cut-off	NO. of patients	Subtype	HR of DFS	HR of DFS (95% CI)	HR of OS	HR of OS (95% CI)
Agoff[7]	2003	America	5%	69	ER neg	0.33	0.1-1.0	NA	NA
Schippinger[8]	2006	Austria	10%	232	all	0.803	0.567-1.138	NA	NA
Gonzalez[9]	2008	Spain	1%	111	all	NA	NA	0.46	0.23-0.93
Luo[10]	2010	China	1%	137	TNBC	0.394	0.110-1.404	1.72	0.891-2.132
Castellano[11]	2011	Italy	10%	859	ER pos	0.444	0.258-0.765	0.135	0.054-0.337
Yu[12]	2011	China	10%	327	ER pos	0.309	0.192-0.496	NA	NA
Park[13]	2011	Korea	10%	931	all	NA	NA	NA	NA
				672	ER pos	0.654	0.429-0.997	0.647	0.375-1.119
				259	ER neg	1.163	0.061-2.249	1.451	0.701-2.965
Hu[14]	2011	America	1%	1467	all	NA	NA	0.96	0.69-1.34
				1164	ER pos	NA	NA	0.68	0.47-0.99
				303	ER neg	NA	NA	1.59	0.94-2.68
Tang[15]	2012	China	10%	127	TNBC	0.396	0.110-1.452	1.657	0.907-2.032
He[16]	2012	China	5%	287	TNBC	0.4	0.20-0.79	0.47	0.23-0.94
Park[17]	2012	Korea	10%	614	ER pos	0.111	0.013-0.961	0.135	0.015-1.208
This study	2013	China	1%	109	all	0.244	0.068-0.876	0.188	0.041-0.855

DFS, disease free survival; OS, overall survival; HR, hazard ratio; CI, confidence interval ; TNBC, triple negative breast cancer.

**Figure 1:**
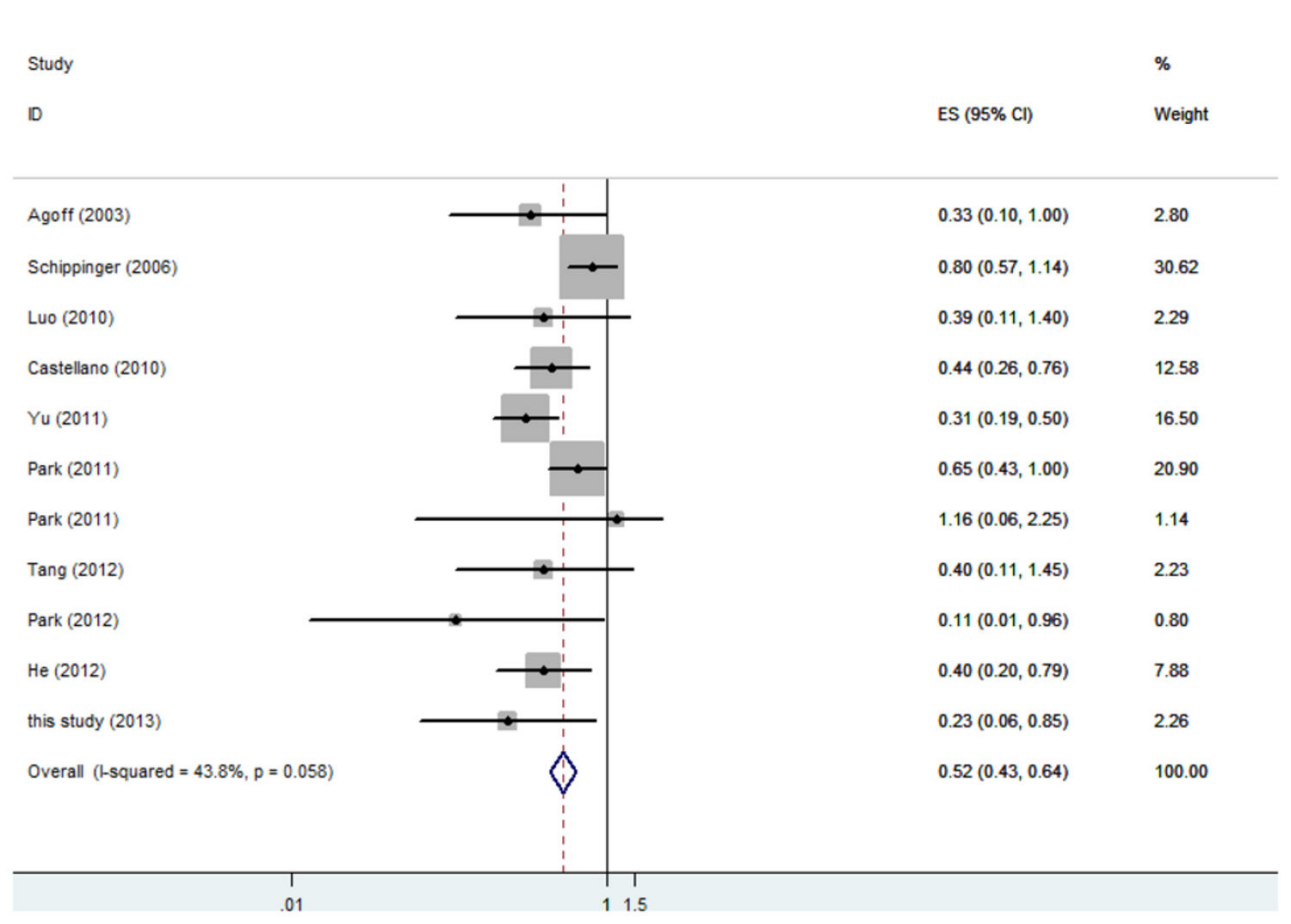
Forest plot (fixed-effects model) of 11 studies assessing the effect of AR expression on DFS in patients with breast cancer. The width of horizontal line represents 95% CI of the individual studies, and the grey boxes represents the weight of each study. The diamond represents the overall summary estimate. The unbroken vertical line was set at the null value (HR = 1.0).

**Figure 2:**
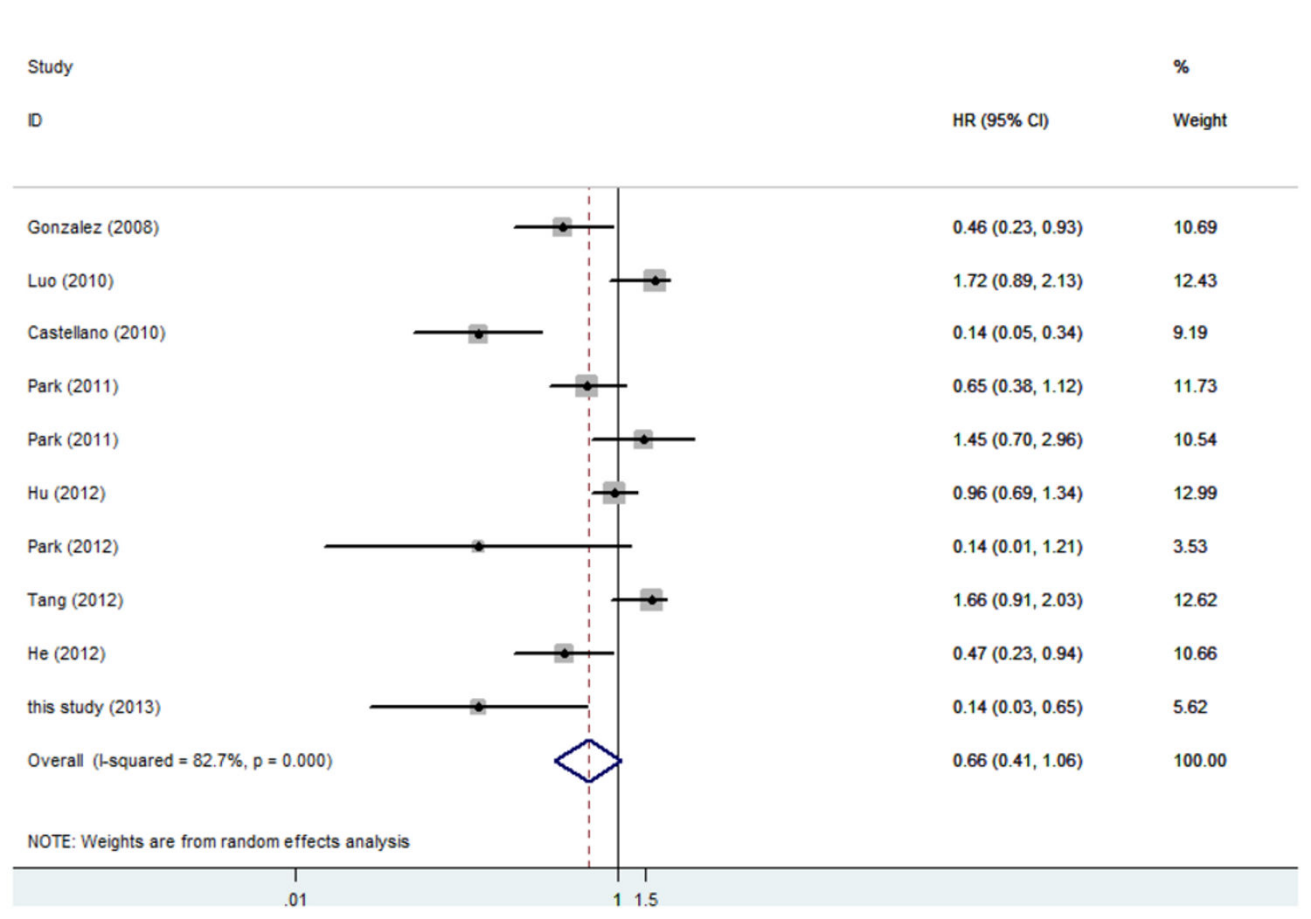
Forest plot (random-effects model) of 10 studies assessing the effect of AR expression on OS in patients with breast cancer. The width of horizontal line represents 95% CI of the individual studies, and the grey boxes represents the weight of each study. The diamond represents the overall summary estimate. The unbroken vertical line was set at the null value (HR = 1.0).

**Figure 3:**
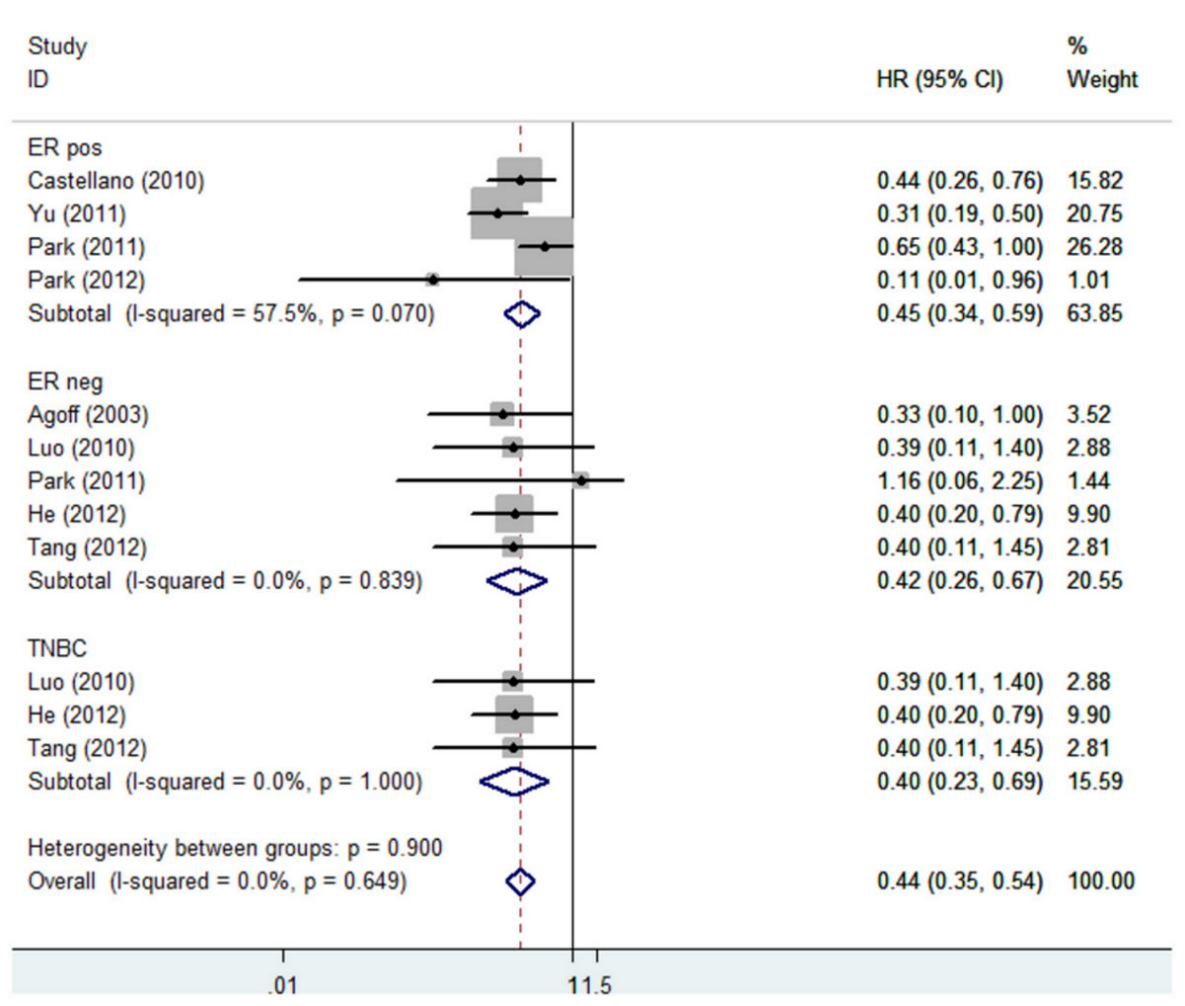
Forest plot (fixed-effects model) of 8 studies assessing the effect of AR expression on DFS in different subtype of breast cancer patients. The width of horizontal line represents 95% CI of the individual studies, and the grey boxes represents the weight of each study. The diamond represents the overall summary estimate. The unbroken vertical line was set at the null value (HR = 1.0).

**Figure 4:**
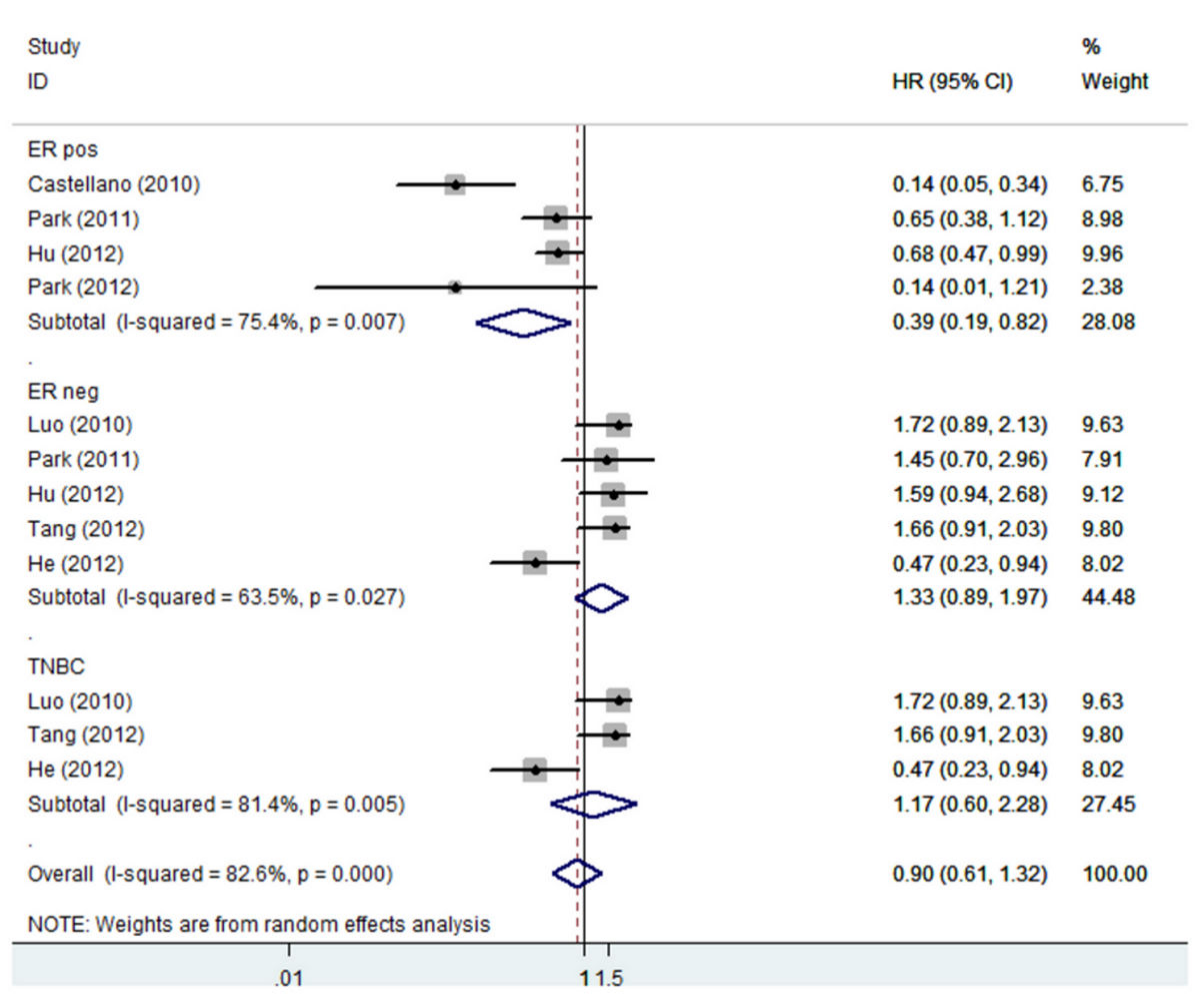
Forest plot (random-effects model) of 7 studies assessing the effect of AR expression on OS in different subtype of breast cancer patients. The width of horizontal line represents 95% CI of the individual studies, and the grey boxes represents the weight of each study. The diamond represents the overall summary estimate. The unbroken vertical line was set at the null value (HR = 1.0).

The overall rate of AR expression in these studies was 65.2%. The combined HR of DFS for all 12 eligible studies was 0.52 (95% CI 0.43–0.64) (Figure 1), indicating that AR expression in breast tumors was an indicator of low risk of recurrence. The HR of OS for all studies was 0.66, but it was not statistically significant (Figure 2). In subgroup analysis, the HR of DFS was 0.45 (95% CI 0.34–0.59) for ER positive patients, 0.42 (95% CI 0.26–0.67) for ER negative patients, and 0.40 (95% CI 0.23–0.69) for patients with triple negative breast cancer (TNBC) (Figure 3). The expression of AR in patients with ER positive breast cancer could also predict OS benefit, and the HR was 0.39 (95% CI 0.19–0.82); but the same was not predictable in ER negative and TNBC subgroups (Figure 4).

## Discussion

Various combined-modality therapies, which are in use in the recent days such as surgery, endocrine therapy, chemotherapy, radiation therapy, and targeted therapy, have improved the outcomes in patients with breast cancer. However, metastasis and recurrence are considered major contributors to treatment failure. Current knowledge of etiopathology, biology, and treatment protocols of breast cancer has benefited from the simultaneous analysis of multiple biomarkers, such as ER, PR, HER2, and Ki67. These four markers are essential in identifying a high-risk phenotype and determining the most efficient therapeutic strategies. However, since breast cancer is a complex and heterogenetic disease, these markers could not cover all disease features. Therefore, besides ER, PR, HER2 and Ki67, it is important to find out new markers with predictive value for survival of patients with breast cancer.

In the past decades, androgens have been identified to improve the efficacy of hormonal treatment and have been used to treat advanced breast cancer; however, their use has declined with the advent of tamoxifen[18]. Androgens and AR may have some important roles in breast cancer. Some studies have examined and indicated that androgen acts through AR in carcinoma cells and play important roles in biology and clinical behavior of breast cancer model systems and cell lines[19,20]. AR, commonly expressed in breast cancer tissues, has been reported as a biomarker to understand the prognosis of breast cancer. However, some clinical studies have reported that AR could not improve the survival. Hence, there is no consensus on the association between AR expression detected by IHC and good survival in patients with breast cancer. The role of AR on survival in breast cancer patients is not very clearly known until now. 

To observe the correlation between AR expression and DFS or OS in patients with breast cancer, the present study retrospectively examined the role of AR evaluated by IHC in breast cancer outcomes and stratified the published researches in a meta-analysis later. Study results have shown that AR is a good marker both for lower risk of recurrence and longer overall survival. The risk of metastasis and death in AR positive patients was significantly lower than in AR negative patients. However, similar results were not obtained in the subgroup analysis, which might probably be due to the small sample size used. 

Meta-analysis is a combination of several studies, and it is less influenced by individual findings from a single study. Since meta-analysis can help to summarize studies on specific topics, the current work also involved a meta-analysis, which compared AR positive versus AR negative expression for DFS and OS in patients with breast cancer. To the best of our knowledge, this is the first comprehensive and detailed meta-analysis, which revealed the prognostic role of AR in breast cancer. The present analysis, combined 12 independent studies, which included 5,270 patients with breast cancer. The results revealed that AR expression could predict lower risk of relapse in patients with breast cancer. In subgroup analysis, AR could also predict better DFS in patients with ER positive, ER negative, and TNBC types. The expression of AR could also predict better OS in patients with ER positive breast cancer.

This systematic review with meta-analysis had to address heterogeneity issues. A moderately significant heterogeneity was observed among the 12 studies. A well-standardized technique is very important to evaluate biological markers. However, there remained some limitations in this meta-analysis. In the studies included, the antibodies used in detecting AR expression were not the same. The definition of cut-off value was also different and varied from 1% to 10%. The heterogeneity also could be explained by the different molecule type of tumors and different disease characteristics. 

Some trials had to be excluded from the meta-analysis, because they did not provide sufficient data on survival. Among them, 5 excluded studies, reported significant associations between AR expression and survival, but they did not have the values of HR and 95% CI of survival[21-25]. 

Several other limitations of these studies could not be ignored. Results of multivariate survival analysis reported in the articles were included in the meta-analysis; if these data were not available, univariate data were extracted instead. Another limitation was from DFS and progression free survival (PFS). Schippinger’s research was focused on the relationship between AR and PFS in the metastatic patients, however the present analysis combined the results of PFS together as DFS[8]. Park’s study separately reported the HR and 95% CI of AR in ER positive and ER negative patients. However, HR and 95% CI was not reported for all the patients studied. This made us to analyze this study as two separate studies for the meta-analysis[13]. 

Since the review was limited to the published scientific literature, a potential impact of publication bias could not be ruled out. Publication bias is a major concern for meta-analysis, because positive results tend to be accepted by journals, while negative results are often rejected.

## Conclusion

Meta-analysis suggested that AR expression was associated with low risk of recurrence of breast cancer. It could be used to identify the low-risk patients earlier and guide clinical decisions. AR expression and breast cancer OS was dependent on ER expression. The influence of AR was significant for the OS among the ER positive patients. Since all these articles are retrospective in nature, well-designed prospective studies are recommended.

## Supporting Information

Figure S1 Immunohistochemical analysis of AR (high-power field *400 magnification). A. Negative for AR. B. positive for AR.
(TIF)

Table S1 **Patient characteristics based on AR expression.**
(DOCX)

Checklist S1 **PRISMA 2009 checklist.**
(DOC)

Flow Diagram S1 **PRISMA 2009 Flow Diagram.**
(DOC)
