# Supplementary figures and images for: The Impact of Androgen Receptor Expression on Breast Cancer Survival: A Retrospective Study and Meta-Analysis
Source: PLoS One. 2013 Dec 4;8(12):e82650. doi: 10.1371/journal.pone.0082650 (PMC3853592; doi:10.1371/journal.pone.0082650)

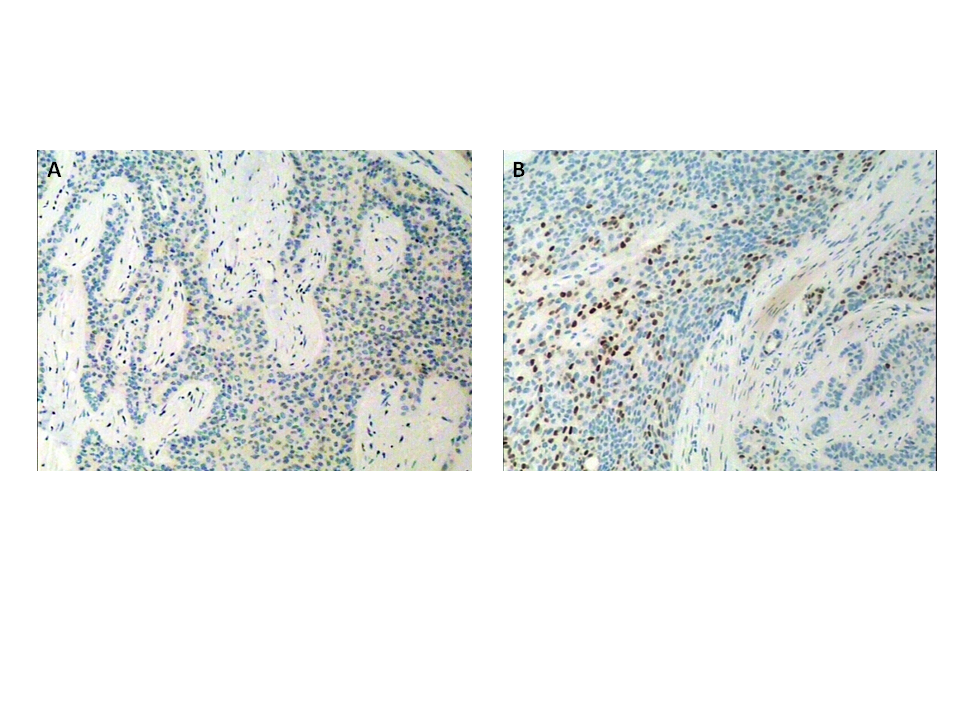

Supplement: Figure S1 — Immunohistochemical analysis of AR (high-power field *400 magnification). A. Negative for AR. B. positive for AR. (TIF) [file pone.0082650.s001.tif]
