# Supplementary material for: The Impact of Androgen Receptor Expression on Breast Cancer Survival: A Retrospective Study and Meta-Analysis
Source: PLoS One. 2013 Dec 4;8(12):e82650. doi: 10.1371/journal.pone.0082650 (PMC3853592; doi:10.1371/journal.pone.0082650)
Supplement: Table S1 — Patient characteristics based on AR expression. (DOCX) [file pone.0082650.s002.docx]

Table S1 Patient characteristics based on AR expression

|  | AR negative  n=57 (%) | AR positive  n=52 (%) | p value |
| --- | --- | --- | --- |
| Age |  |  |  |
| >50 years | 45 (78.9) | 35 (67.3) |  |
| ≤ 50years | 12 (21.1) | 17 (32.7) | 0.197 |
| Tumour size |  |  |  |
| > 2cm | 26 (45.6) | 29 (55.8) |  |
| ≤ 2cm | 31 (53.4) | 23 (44.2) | 0.340 |
| Lymph node status |  |  |  |
| Metastasis | 24 (42.1) | 12 (23.1) |  |
| No metastasis | 33 (57.9) | 40 (76.9) | **0.043** |
| ER status |  |  |  |
| Positive | 40 (70.2) | 39 ( 75.0) |  |
| Negative | 17 (29.8) | 13 (25.0) | 0.669 |
| PR status |  |  |  |
| Positive | 33 (57.9) | 34 (65.4) |  |
| Negative | 24 (42.1) | 18 (34.6) | 0.439 |

AR, androgen receptor; ER, estrogen receptor; PR, progesterone receptor; HER2, human epidermal growth factor receptor 2.
