# Supplementary material for: The Impact of Androgen Receptor Expression on Breast Cancer Survival: A Retrospective Study and Meta-Analysis
Source: PLoS One. 2013 Dec 4;8(12):e82650. doi: 10.1371/journal.pone.0082650 (PMC3853592; doi:10.1371/journal.pone.0082650)
Supplement: Flow Diagram S1 — PRISMA 2009 Flow Diagram. (DOC) [file pone.0082650.s004.doc]

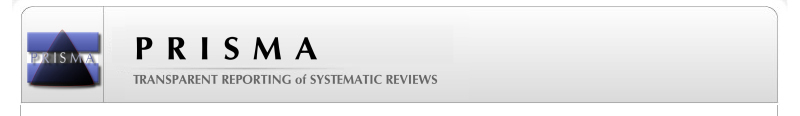
**PRISMA 2009 Flow Diagram**

**Screening**

**Included**

**Eligibility**

**Identification**

Records identified through database searching
(n = 1376 )

Additional records identified through other sources
(n = 0 )

Records after duplicates removed
(n = 790)

Records screened
(n = 54 )

Records excluded
(n = 32 )

Full-text articles assessed for eligibility
(n = 22 )

Full-text articles excluded, with reasons
(n = 6 )

Studies included in qualitative synthesis
(n = 16 )

Studies included in quantitative synthesis (meta-analysis)
(n = 11 )
